# The effectiveness of fatigue on repositioning sense of lower extremities: systematic review and meta-analysis

**DOI:** 10.1186/s13102-024-00820-w

**Published:** 2024-02-05

**Authors:** Parisa Sayyadi, Hooman Minoonejad, Foad Seidi, Rahman Shikhhoseini, Ramin Arghadeh

**Affiliations:** 1 Department of Sports injury and biomechanics, Faculty of Sport Sciences and health, University of Tehran, Tehran, Iran; 2 Department of Sports injury and biomechanics, Faculty of Sport Sciences and health, University of Tehran, Iran, Tehran, Iran; 3 Department of Sports injury and biomechanics, Faculty of Sport Sciences and health, University of Tehran, Iran, Tehran, Iran; 4 Department of Corrective Exercise and Sports Injury, Faculty of Physical Education and Sport Sciences, Allameh Tabataba’i University, Tehran, Iran; 5 Department of Sports injury and biomechanics, Faculty of Sport Sciences and health, University of Tehran, Tehran, Iran

**Keywords:** Fatigue, Knee, Proprioception, Sense of position

## Abstract

**Introduction:**

An injury can significantly harm both individual and team performance. One of the most important risk factors for sports-related injuries, especially non-collision injuries, is fatigue. It seems that poor proprioception may play an essential role to impose athletes to further injuries. This systematic review and meta-analysis aimed to examine the effectiveness of fatigue on the repositioning sense of the lower extremity joints.

**Method:**

The electronic databases, including PubMed, Web of Science, Scopus, and Google Scholar were systematically searched from inception to 11January 2024. The obtained records were exported to the EndNote Software version 8. Then, two investigators examined the records independently to find eligible studies based on the inclusion/exclusion criteria. In the case of disagreements, a consequence method was utilized. The quality of the eligible studies was evaluated using the Downs and Black checklist. Comprehensive Meta-Analysis (CMA) software ver. 3 software was used for statistical analysis. Q-test and I^2^ were employed to examine the data homogeneity. In addition, considering the risk of bias, the Funnel Plot and trim-and-fill method were used.

**Results:**

After reviewing the titles and abstracts of 3883 studies found in the selected databases, 43 articles were found to be eligible to include in meta-analyses. The results showed that fatigue led to a significant increase in the active absolute error of the knee (SDM = 0.524, 95% CI = 0.406–0.841), ankle in the horizontal plane (SDM = 0.541, 95% CI = 0.367–0.715), ankle in the sagittal plane (SDM = 0.443, 95% CI = 0.088–0.798), and hip (SDM = 0.988, 95% CI = 0.135–1.841). However, fatigue had no significant effects on the passive absolute error of the knee and ankle in horizontal plane and relative angular error of the knee.

**Conclusion:**

Fatigue can diminish the active joint position sense of the lower extremities and thus may increase the risk of injury by reducing proprioception. Therefore, future research could be conducted to investigate the potential impact of integrated fatigue-mitigating exercises into athletes’ training programs, with the aim of reducing the incidence of sports-related injuries.

**Supplementary Information:**

The online version contains supplementary material available at 10.1186/s13102-024-00820-w.

## Introduction

Proprioceptive information describes the degree and change in muscle length and tension, joint angle, and skin tension by collecting peripheral sensory inputs through mechanoreceptors in ligaments, muscles, and skin [1]. muscle receptors provide the majority of afferent information, it is expected that changes in muscle length and tension will affect the accuracy of joint position sense [2, 3].

Joint position sense is a component of proprioception, and the alteration in joint position sense is a significant factor in joint coordination, muscle stiffness, movement integration, and movement disorders [4]. In sports, joint position sense accuracy is extremely important because it correlates highly with skill accuracy and injury risk [5].

Participation in sports is associated with the risk of injury. A sports injury can cause pain, disabilities, and withdrawal from sport [6]. A sprained ankle is one of the most prevalent musculoskeletal injuries [7, 8]. Several studies have observed that sports that involve sudden stopping and cutting movements, such as soccer, have the highest incidence of these types of injuries [9, 10] . In addition to the financial costs associated with such injuries, they can also result in significant time loss due to injury, delayed return to activity, and long-term disability in up to 60% of injured athletes [11]. Symptoms induced from frequent ankle instability can be threatening to health and lead to less active lifestyle as well [12].

In addition to ankle injuries, knee injuries, particularly anterior cruciate ligament (ACL) injuries, are among the most common, and they typically occur without external contact other than ground contact [5]. The majority of these injuries result from non-contact incidents during sudden deceleration or landing maneuvers [13]. It has been observed that females have a higher incidence of ACL injuries [14]. Since these injuries are not collision-induced, it seems that preventative methods may help athletes to decrease the risk of injuries.

On the other hand, evidence suggests that knee injury is associated with a diminished repositioning sense, possibly due to a decrease in proprioceptive inputs from mechanoreceptors [15]. Lower extremitiy injuries, such as ACL tears, can result from inadequate neuromuscular responses to sensory information [13]. Consequently, joint position sense is crucial for preventing these injuries [16]. Furthermore, fatigue may adversely affect joint position sense [17]. Fatigue-induced alterations in neuromuscular control of the lower extremities and dynamic stability [18] may cause most injuries to occur in the final third of training or competition [19]. These negative effects on neuromuscular control and dynamic stability of the lower extremities may result from changes in joint position sense [20].

Fatigue has been frequently reported to disrupt motor control, leading to neuromuscular activation delay and an increase in torque and shear forces, thereby endangering joint stability [21]. Additionally, it increases postural sway and impairs the capacity to maintain balance. Researchers have also indicated that a decrease in proprioception can lead to impairment in some parameters such as reaction time, postural control, and balance [22, 23]. Studies indicate that most ankle sprains occur in the last one-third or two-thirds of a race, suggesting that fatigue alters the neuromuscular control of the ankle and the ankle’s ability to maintain dynamic stability [24]. Peroneus longus and brevis play a crucial role in preventing ankle sprains. These muscles prevent sudden ankle inversion [25]. An increase in the delayed latency of the peroneal reflex has been evidenced in patients with chronic, acute ankle instability caused by recurrent foot sprains [26].

Moreover, neuromuscular fatigue is a risk factor for cruciate ligament injury [27]. Due to delayed muscle activation and disruption of the excitation-contraction process, studies show that fatigue causes an increase in reaction time [28, 29]. The long reaction time of the hamstring impairs the muscle’s ability to quickly stabilize the knee when the knee is loaded in sports and increases the risk of knee injury [30].

Numerous studies have examined the effect of fatigue on lower extremity position sense. Some of them have evidenced that fatigue can significantly impair the repositioning sense of the lower extremities [2, 3, 31, 32] whileothers have repositioning not [22, 33–35]. Thus, we aimed to conduct a systematic review with meta-analysis to combine existing results from various studies on the possible effects of fatigue on repositioning sense in knee, ankle, and hip joints.

## Methodology

### Search strategy

This systematic review was a-priori registered and executed according to Cochrane guidelines and the PRISMA-2020 checklist (Preferred Reporting Items for Systematic Reviews and Meta-Analyses). The PROSPERO registration number CRD42021274701 was taken before starting the searches. The search strategy was used to extract all eligible articles..To identify relevant articles, we systematically searched Scopus, Web of Science, and PubMed from database inception to 11 January 2024 using a combination of terms relevant to ‘lower extremity’, ‘position sense’ and ‘fatigue’. Google Scholar, Elmnet, Magian and SID was also searched. In addition, references of relevant articles were scrutinized manually to identify additional potentially eligible literature.

### Search keyword

(hip OR knee OR ankle OR foot OR feet OR “lower extremity” OR “lower limb” OR “lower-limb” OR “lower-extremity”) AND (proprioception OR “position sense” OR “reposition error” OR reposition* OR “sense of position” OR neuromuscular) AND (fatigue* OR lassitude OR tiredness OR exhaustion)

### Eligibility criteria

Articles in English and Persian were deemed eligible for inclusion if they had examined the effect of fatigue on the repositioning sense of lower limb and measured variables related to repositioning sense. Studies on individuals with neurological disorders, ligament laxity, arthritis, and sensory or coordination issues were excluded. Studies with insufficient information, data from conference articles, and published abstracts without full-text papers were also removed.

### Study selection

First, the search strategy was performed in the respective databases. Records were entered in the Endnote file by preserving the names of authors, titles, and abstracts of sources. After eliminating duplicates, Two independent reviewers (PS, RA) screened all potentially relevant titles and abstracts for eligibility and disagreements were discussed and resolved by third reviewers (R.S).

### Data extraction and quality assessment

Two reviewers (P.S, R.A) assessed all selected articles independently for risk of bias using the Downs and Black checklist to evaluate the quality of the articles [36] (Supplementary material 1), and any discrepancies were resolved by a third reviewer (R.S). The validity and reliability of this checklist have been established previously [36]. Similar to previous review and meta-analysis articles, this study also used 22 items from this checklist [37]. The total score from this checklist was presented as follows: more than 65% as low risk of bias, and less than 65% as high risk of bias [38]. Based on previous studies, we changed the last item of the checklist from 0 to 5 to 0–1 [39]. 22 articles were assessed low risk of bias and 21 articles were assessed high risk of bias. The following data were extracted from each included study: first author’s name and year of publication, sample size, participants’ demographic information (i.e. age, sex), training characteristics (i.e. type, duration, and others), data collection instruments, and main reults (Supplementary material 2).

### Data analysis

The required data (standard deviation, mean of pre-and post-tests, *P*-values, sample size, and, if possible, standard deviation and mean difference) were extracted from articles that met the inclusion criteria. Comprehensive Meta-Analysis 3.3 software was utilized. A random effects model was used to analyze the data. The standad difference in means and 95%confidence interval (CI) were used to report the overall effect size.heterogenity of the studies was assessed using I^2^,with values at 25, 50, and 75% considered as low, medium, and high heterogeneity [40], respectively and Q-test, with a significant level of < 0.05 [40]. Egger^,^s test egression test was used to evaluated the statistical significant of publication bias. *P*-value < 0.05 were considered significant of publication bias [41]. Also A funnel plot was utilized to evaluate the risk of bias. If a potential risk of bias was observed, the trim-and-fill method was used To determine how many studies should be imputed for symmetrical distribution of the effect sizes. The P-value for significance of the pooled effect analyses was set at < 0.05.

## Results

### Search result

**Fig. 1:**
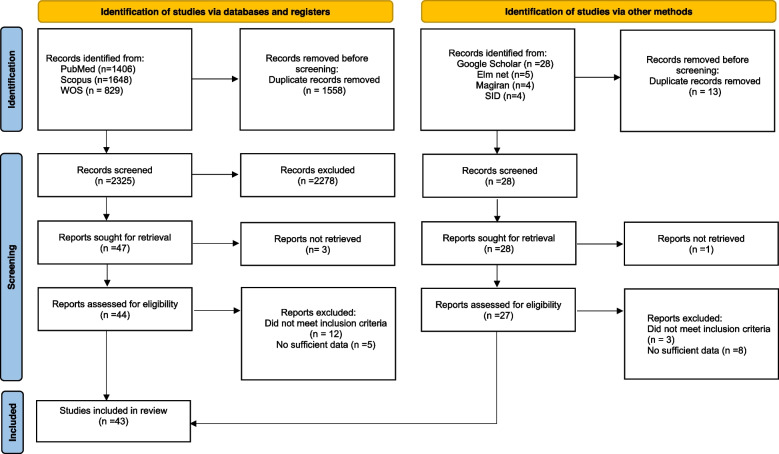
Search and selection of studies for systematic review according to PRISMA guidelines

In total, 3,883 articles were found in the selected databases. After removing duplicates, 2325 articles that were then screened for inclusion. After reviewing the abstracts and titles, 44 articles were included. The full texts of the articles were then carefully reviewed, and 27 eligible articles were included in the study, Also, 16 studies were included from other sourses including Google scholar, Elm net, Magiran and SID, and finally the number of studies included in the meta-analysis was 43 (Fig. 1). For more accuracy, the articles were first classified into knee, ankle and hip joints. Then, in each section, the articles related to absolute and relative error were examined separately in two groups. Active and passive absolute error as well as relative error.

## Description of the selected variables

### Measurement of joint reposition sense

Various measurement techniques have been used to measure joint reposition sense. Eighteen studies employed isokinetic dynamometer [22, 29, 33, 34, 42–55], five studies used photogrammetry [2, 3, 35, 56, 57], two studies used goniometers and photogrammetry [58, 59], two studies used an electrogoniometer [32, 60], three studies used a potentiometer [61–63], four studies used a goniometer [43, 64–66], two studies used a joint position sense device [67–69], three studies used an inclinometer [70–72], one study used an Air Splint [73], one study used an optotrak [74], and two study used a self-design device [31, 69]. In addition, the following studies employed two types of measurement error: 36 studies investigated the absolute angular error [2, 3, 22, 29, 31–35, 42–51, 53–59, 63, 65, 67–74] (absolute difference between the target position and the estimated one), while 12 studies investigated the relative angular error [2, 3, 31, 42, 46, 50, 56, 57, 61, 62, 64, 72] (difference between the target position and the repositioning angle, taking the direction of the difference into account). Thirteen studies examined passive joint reposition sense [22, 29, 31, 33, 34, 43, 47, 48, 53, 54, 67, 68, 75], while 34 examined active joint reposition sense [2, 3, 29, 31, 32, 35, 42–46, 48–53, 55–65, 69–74, 76].

### Fatigue protocol

The studies have employed various protocols to induce fatigue. Two studies [22, 59] used a bicycle, and some studies used local muscle fatigue using an isokinetic dynamometer [2, 29, 31, 33–35, 42–46, 50–55, 62, 63, 68, 69, 73, 75, 76]. One study utilized volleyball games [3], three used plyometric exercises [48, 56, 65], one used squats [32], and one employed stairs [61]. In addition, one study used a dynamometer [72], one study used handball game [70], two study used soccer games [53, 57], and one study used running [47] to induce fatigue. In addition, standing on the toes for as long as possible (Gastrocsoleus muscle) [60, 67], modified protocol Bangsbo pseudo-football [49], karate [64], quadriceps muscle fatigue with weights [71], Sorenson [58], lumbar flexor fatigue [52], shuttle test [66] and hip abduction exercises [74] were used to induce fatigue in other studies. Local fatigue was considered in studies examining local and global fatigue in the same group.

### Joints

Three joints of knee [2, 3, 22, 31, 32, 34, 35, 42, 44–46, 48, 50–52, 55–59, 61, 62, 64–66, 69–73], ankle [16, 29, 33, 43, 47, 49, 53, 54, 60, 63, 67, 68, 75], and hip [58, 70, 74] joints were investigated in these studies.

### Angle

In the knee joint, 24 studies examined the joint position sense in the mid-range [2, 3, 22, 31, 32, 34, 35, 42, 45, 46, 48, 50–52, 55–58, 61, 62, 64, 65, 69, 71], four studies in the initial knee flexion range [59, 66, 73, 74], and two studies in the end knee flexion range [44, 72]. Additionally, the angle of the test had not been specified in one study [70]. Some studies examined knee repositioning sense in different knee angles. As the functional range of the knee angle lies between 45 and 60 degrees of flexion, the angles closer to this range were chosen for meta-analyses.

In the ankle joint, eight studies measured the reposition sense within 10–20 degrees of inversion [29, 33, 47, 49, 53, 54, 68, 75], and four studies within 15–21 degrees of plantar flexion [17, 43, 60, 63, 67]. Moreover, in studies that had examined multiple angles, angles closer or within the mid-range were considered.

## Data synthesis

### The active absolute angular error of the knee

**Fig. 2:**
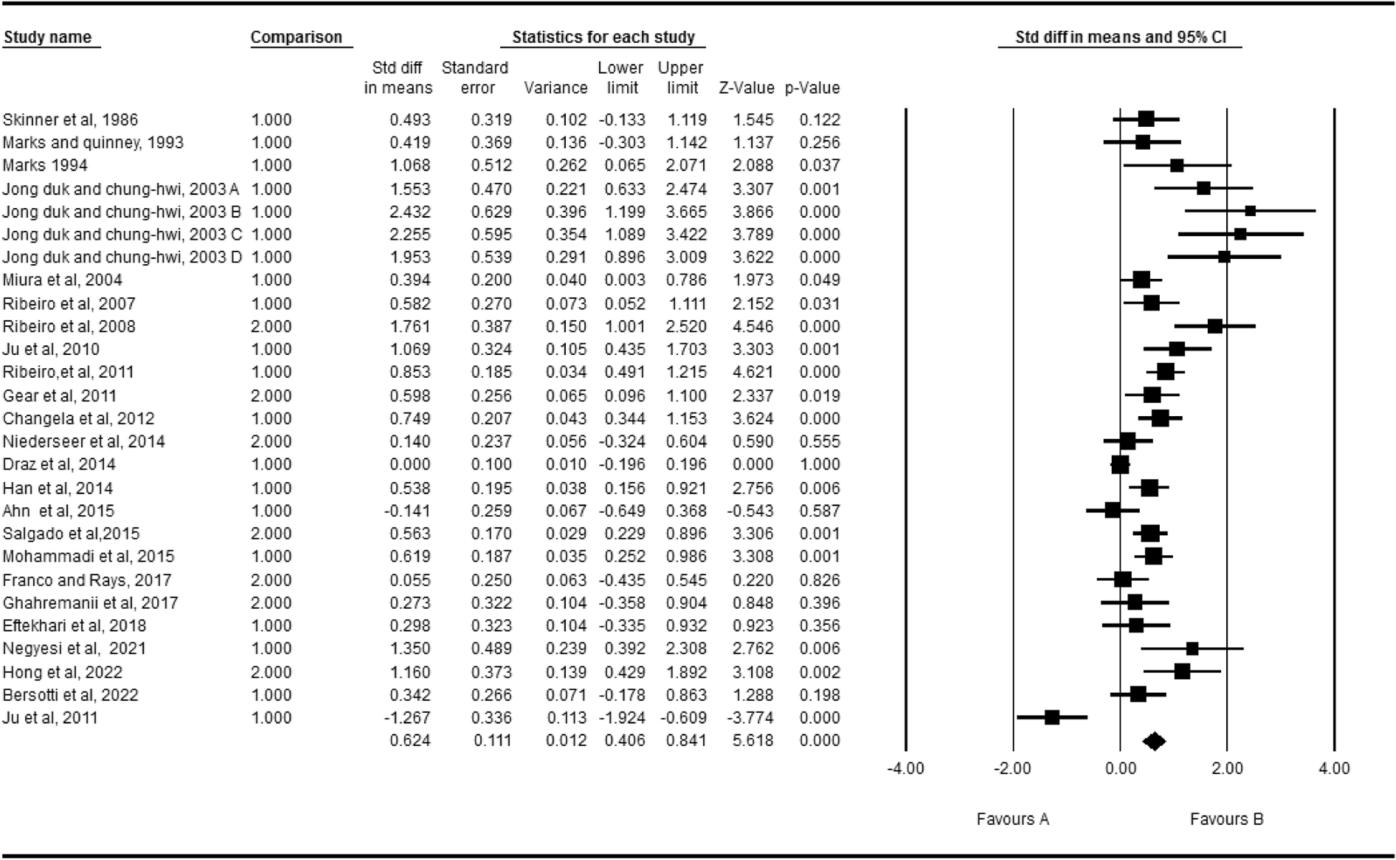
Forest plot of the effect of fatigue on active absolute error angle

**Fig. 3:**
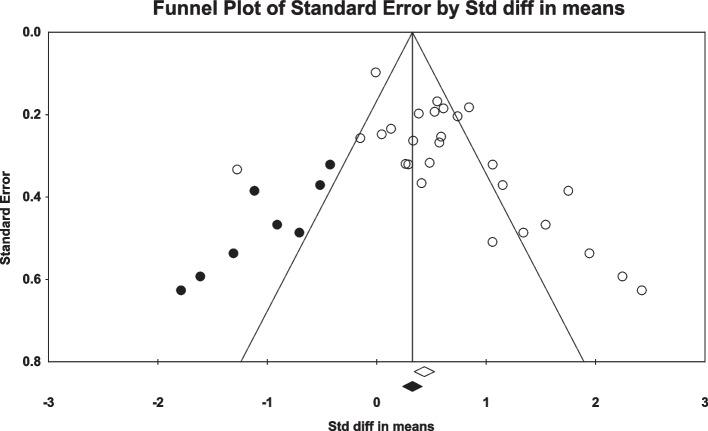
Funnel plot of studies worked on active absolute error angle. Hollow circles: observed studies, gray filled circles: random imputed studies

The effects of fatigue on the active absolute angular error of the knee are depicted in Fig. 2. Twenty four studies investigated the effect of fatigue on the active absolute angular error of the knee [2, 3, 31, 32, 35, 42, 44–46, 48, 50–52, 55–59, 65, 69–73]. One study had four independent groups all of them included in the meta-analyses. In total, 536 individuals participated in these studies. According to the meta-analyses, fatigue can significantly affect the active absolute angular error of the knee (SDM = 0.524, 95% CI = 0.406–0.841). The Q-test and I^2^ test results indicated significant heterogeneity across studies (*P* = 0.001, I^2^ = 78.57). Egger’s test and Funnel plot showed publication bias is statistically significant (P = 0.001). However, Duval and Tweedie’s trim and fill method imputed eight potential missing studies to the left side of the plot although the result of pooled effect did not change significantly showing the robustness of the results (Fig. 3).

### The passive absolute angular error of the knee

**Fig. 4:**
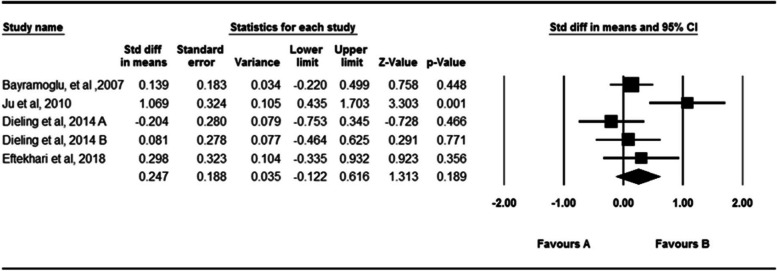
Forest plot of the effect of fatigue on passive absolute error angle

Four studies investigated the effect of fatigue on knee passive absolute angular error [22, 31, 34, 48] (Fig. 4). In these studies, 81 participants participated. According to the analysis of the results, fatigue has no significant effect on passive absolute knee angular error (SDM = 0.247, 95% CI = − 0.122-0.616 The Q-test and I^2^ results indicated significant heterogeneity across studies (*P* = 0.046, I^2^ = 58.62. The Egger’s test and Funnel plot did not indicate publication bias (*P* = 0.52). (Supplementary material 3).

### The active relative angular error of the knee

**Fig. 5:**
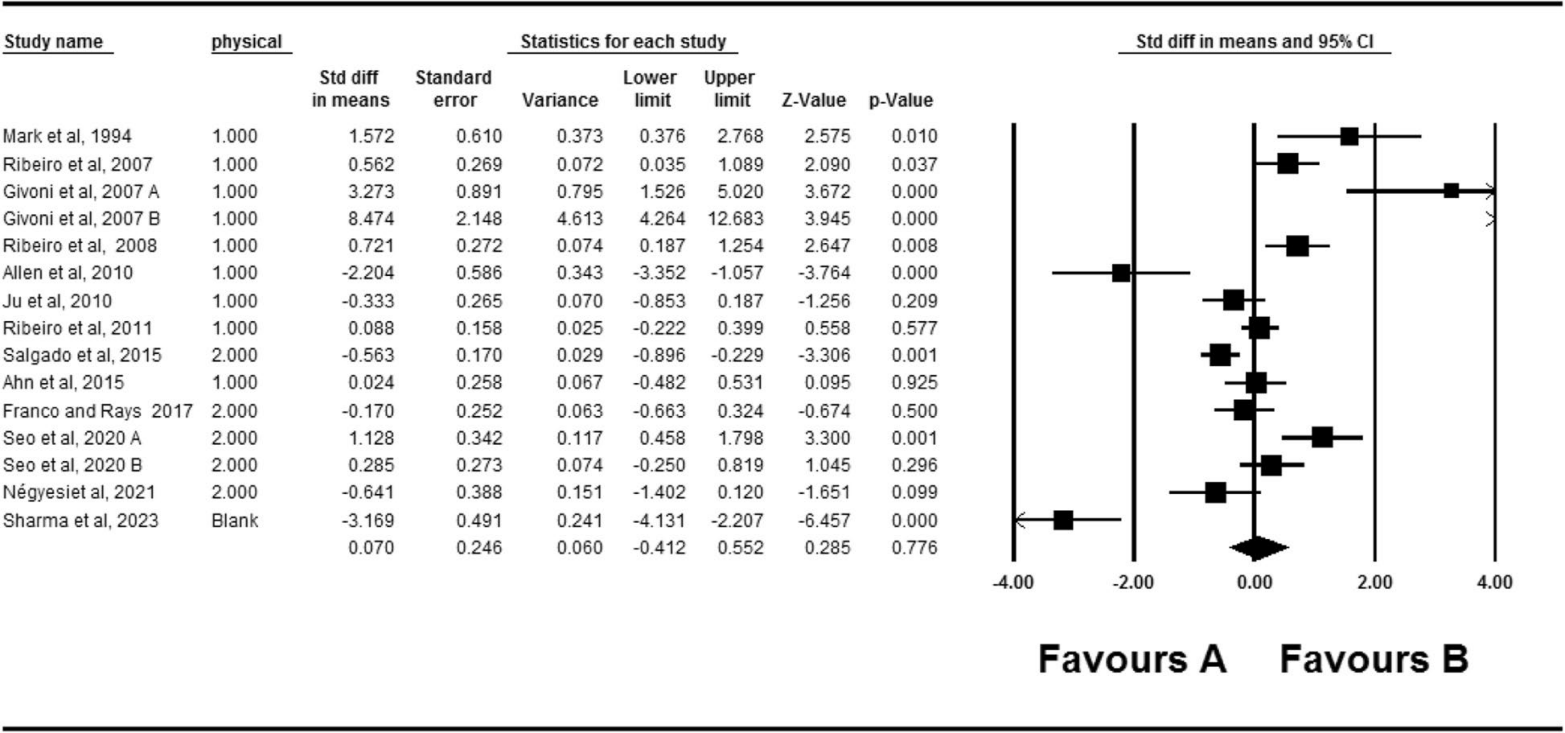
Forest plot of the effect of fatigue on active relative error angle

Thirteen studies investigated the effect of fatigue on active relative angular error [2, 3, 31, 42, 46, 50, 56, 57, 61, 62, 64, 72] (Fig. 5). Two independent groups were utilized in two studies. 237 participants took part in these studies. The results revealed that fatigue has no significant effect on active relative angular error (SDM = 0.070, 95% CI = -0.412–0.552). In addition, The Q-test and I^2^ test results indicated significant heterogeneity across studies (*P* = 0.001, I^2^ = 89.291). Moreover, The Egger’s test and Funnel plot did not indicate publication bias (*P* = 0.44). (Supplementary material 3).

### The active absolute angular error of the ankle in the horizontal plan

**Fig. 6:**
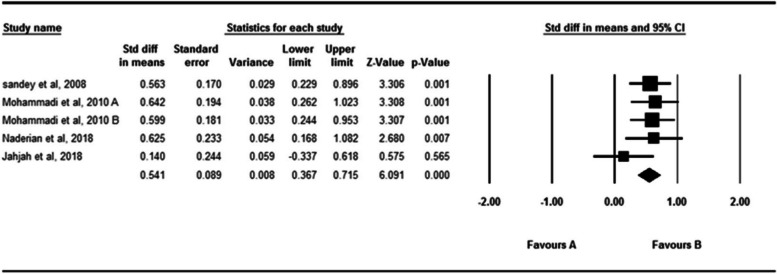
Forest plot of the effect of fatigue on active absolute error angle

Figure 6 indicates the effect of fatigue on the active absolute angular error of the ankle in the horizontal plane. 4 studies examined the effect of fatigue on the active absolute angular error of the ankle [29, 49, 53, 68]. 147 participantsparticipated in these studies. As shown by the analysis of the data in these studies, fatigue significantly affects the active absolute angular error of the ankle (SDM = 0.541, 95% CI = 0.367–0.715) The Q-test and I^2^ test results didn’t indicate significant heterogeneity across studies (*P* = 0.521, I^2^ = 0.001). The Egger’s test and Funnel plot didn’t indicated publication bias (*P* = 0.31). (Supplementary material 3).

### The passive absolute angular error of the ankle in the horizontal plane

**Fig. 7:**
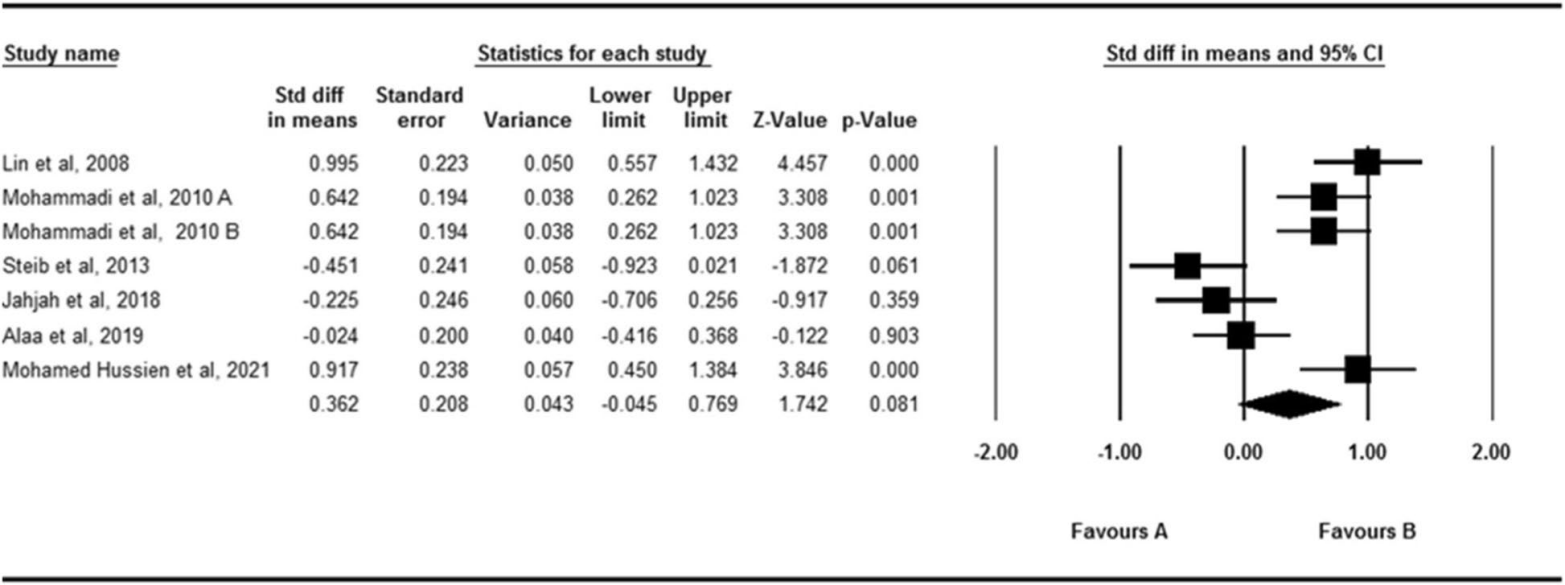
Forest plot of the effect of fatigue on passive absolute error angle

The effect of fatigue on the passive absolute angular error of the ankle in the horizontal plane is shown in Fig. 7. Six studies studied the effect of fatigue on the passive absolute angular error of the ankle [29, 33, 47, 53, 54, 68]. 180 subjects participated in these studies. According to the analysis of the data in these studies, fatigue did not affect the passive absolute angular error significantly (SDM = 0.362, 95% CI = − 0.045- 0.769). The Q-test and I2 test results indicated significant heterogeneity across studies (*P* = 0.001, I^2^ = 84.36). The Egger’s test and Funnel plot didn’t indicated publication bias (*P* = 0.051). (Supplementary material 3).

### The active absolute angular error of the ankle in the sagittal plane

**Fig. 8:**
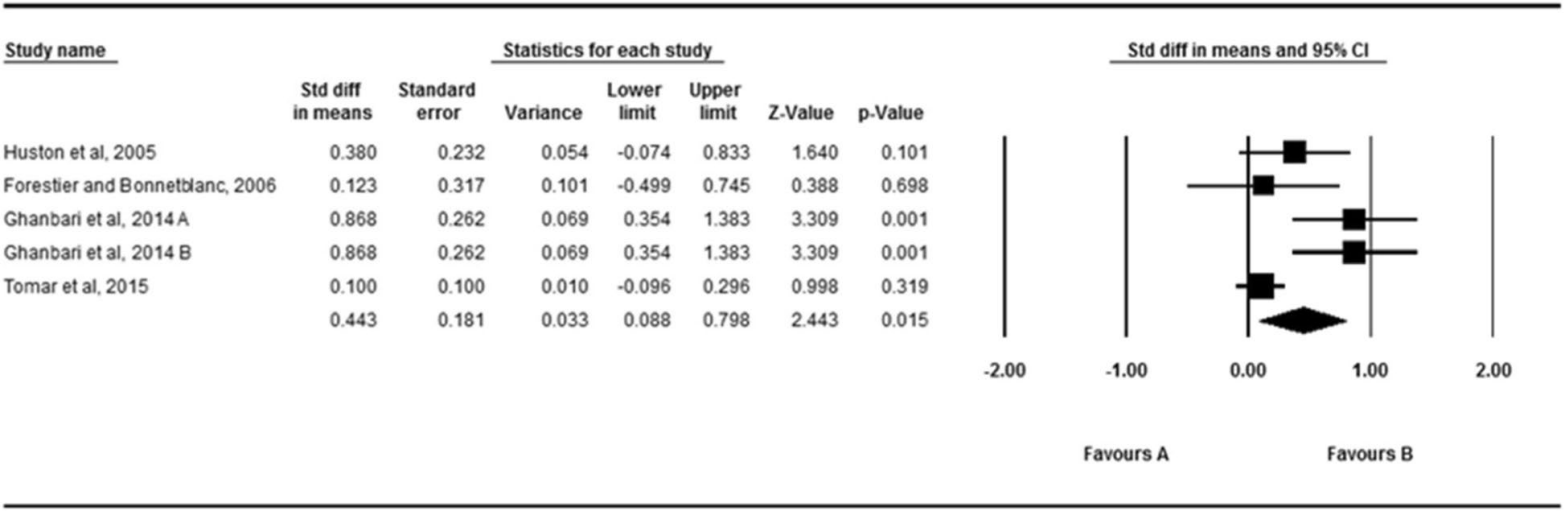
Forest plot of the effect of fatigue on active absolute error angle

The effect of fatigue on the active absolute angular error of the ankle in the sagittal plane is shown in Fig. 8. 4 studies examined the effect of fatigue on the passive absolute angular error of the ankle [43, 60, 63, 67]. A total of 150 subjects participated in these studies. According to the analysis of the data in these studies, fatigue has a significant effect on the active absolute angular error (SDM = 0.443, 95% CI = 0.088–0.798).the I^2^ and Q test were indicated that heterogeneity wasn’t significant (*P* = 0.08, I^2^ = 70.81). The Egger’s and Funnel plot test didn’t indicated publication (*P* = 0.16) (Supplementary material 3).

### The active absolute angular error of the hip

**Fig. 9:**
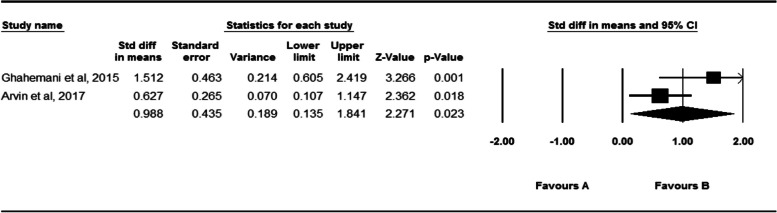
Forest plot of the effect of fatigue on active absolute error angle

Figure 9 indicates the effect of fatigue on the active absolute angular error of the hip. Two studies examined the effect of fatigue on the active absolute angular error of the hip [58, 74]. Twenty-seven subjects participated in these studies. As shown by the data analysis of these studies, fatigue has a significant effect on the active absolute angular error of the hip (SDM = 0.988, 95% CI = 0.135–1.841). I^2^ and Q test were shown that heterogeneity was not significant (*P* = 0.097, I ^2^ = 63.6).

## Discussion

As per findings of this study, fatigue can significantly affectthe active absolute angular error of the knee, ankle, and hip, but not the active relative angular error of the knee or the passive angular error of the knee and ankle in the horizontal plane. Furthermore, fatigue can affectabsolute and relative angular error differently, with only absolute angular error being significantly affected. In addition, only the active but not passive angular error was significantly impacted by fatigue.

The following factors may account for the observed difference between the effects of fatigue on active and passive absolute angular error. The repositioning sense is evaluated either in active or passive movements. Studies have demonstrated that, the active test is more accurate, functional than the passive test, and can maximize muscle inputs [70, 71]. Previous researches have indicated that muscle receptors play the most significant role in the joint repositioning sense [20, 77]. Since a fatigue protocol influences muscle receptors more than joint tissues [20], an active test is more affected by fatigue than a passive test. In addition, muscle activity is reduced during passive movements. Therefore, the fusimotor and the sensory feedback from the muscle spindle are less active, and Ruffini endings as well as skin sensory receptors are primarily responsible for passive movements [78].

The different effect of fatigue on active relative and absolute angular error is an additional significant difference between the variables of our findings. In an absolute angular error, the difference in the angle difference is calculated without considering the direction, which may account for a significant portion of this difference in the result. In contrast, the relative angular error also considers the direction. Because certain errors are recorded as negative numbers while others are reported as positive ones, using relative error can reduce the disparities.

Multiple factors contribute to the detrimental effect of fatigue on proprioception. Fatigue-induced physiological and biochemical changes are one of these crucial factors. In the condition known as local fatigue, nociceptors are activated by the production of muscle metabolic substances such as lactic acid, potassium chloride, bradykinin, and arachidonic acid [79]. These inflammatory and metabolic substances lead to muscle spindle defects [80]. Furthermore, when the anaerobic system is the primary source of energy for the muscle, lactate levels rise rapidly during fatigue, resulting in muscle acidosis and the deterioration of muscle function, which inhibits the activity of mechanoreceptors [81]. Instability of the knee, ligament stretching, and inadequate feedback from mechanoreceptors, which is necessary for triggering the reflexive response of the muscles to maintain joint stability, result from these conditions [82]. Another reason for the negative effect of fatigue on the proprioception may be due to a defect in the central processing of proprioceptive signals caused by central fatigue. The contribution of central nervous systems factor, such as alterations in neurotransmitter levels or brain function in the reduction in voluntary activation of skeletal muscles during physical activity has been termed Central fatigue [83]. Central fatigue leads to defects in the accuracy of motor control, interruptions in the voluntary contraction of muscles to stabilize the joint against applied forces, and thus leads to injury [84].

Studies have also shown that after local isometric muscle contraction, the muscle spindle’s stretch reflex sensitivity increases in contracted muscles [85, 86]. Consequently, if the knee is flexed after an isometric contraction of the quadriceps muscles, the angle perceived is greater than the actual angle. This illusion is caused by the continuous contraction of intrafusal fibers of the muscle spindle, which increases the sensitivity of the muscle spindle’s primary ending [87]. Therefore, the initial position is interpreted as being greater than the actual muscle stretch value, resulting in an overestimation of the target angle. The increase in the error after general load may also be attributable to a deficiency in the central processing of proprioceptive signals [88].

Although funnel plots revealed publication bias, trim-and-fill results demonstrated that imputing a few random studies could not affect the current results. Moreover, significant heterogeneity was observed across eligible studies. The sources of heterogeneity across studies related to fatigue’s effect on the absolute angular error of the knee were investigated using meta-regression and subgroup. Findings indicated that variation in age is the leading cause of heterogeneity across studies. Moreover, meta-regression results did not show significant findings for the angle measurement and sample size. We also divided the studies into two groups of local and general fatigue by using subgroup, but no difference was observed between them, also a distinction is n’t made between studies with a high risk of bias and those with a low risk of bias. Moreover, meta-regression results on the active relative knee angle heterogeneity did not show significant findings for the angle measurement, sample size,and age. Also subgroup results did not difference between local and general fatigue protocol, also between high and low risk of bias studies.

This high heterogeneity may be attributable to various fatigue protocols that have been used in these eligible studies including volleyball, soccer, handball, plyometric exercises, and local exercises of different muscles with Isokinetic dynamometer, stair, bicycle, and treadmill exercises. In addition to the type of exercise, the variation in exercise characteristics, such as intensity and duration, can account for the heterogeneity across studies. This may be caused by thixotropy effects. One factor that may compromise the accuracy of proprioception signaling from muscle spindle is thixotropy. Intrafusal muscle fiber of spindle apparatus behave thixotropcally and this is related to change in resting spindle discharge [89, 90]. When a joint retuned to a reference position after concentric contraction (short- hold) compared with eccentric muscle (long- length) spindle afferent have higher discharge rate in rest that this afferent change may effect on CNS output and joint position sense accurate [91]. Also Studies were finding that Muscle afferent show different responded to fatigue that may be influence by loading condition [91]. Also high heterogeneity may be result from different methods for joint position sense assessment. Stillman and McMeeken (2001) compared JPS assessments in weight-bearing and non-weight-bearing protocols, According to their findings, JPS assessments were more accurate precise when performed in a weight-bearing position [92]. The authors’ explanation is that this may be attributed to the greater stimulation of compressed mechanoreceptors during weight-bearing, but may also be related to the greater ankle dorsiflexion, greater calf complex tension and greater muscle resistance due to the body weight load, which could also contribute to improved accurancy [92]. Also a systematic review study revealed that the intra-rater reliability of JPS assessments varied depending on the assessment method used. Specifically, the review found that JPS assessments using photographs and digital images, as well as a paper model, demonstrated good intra-rater reliability. In contrast, intra-rater reliability was found to be good but variable when electrogoniometry was used, and moderate but variable when assessed using dynamometry or angle motion chairs [93].

This study had some limitations. First, only the acute but not long-term effect of fatigue was investigated. Second, the studies lacked a follow-up assessment. Third,only the repositioning sense was investigated out of the three components of proprioception. In other words, little is known about the sense of force and the sense of kinesthesia. Moreover, only athletes and healthy individuals have been studied, so the results may not be readily generalized to individuals with sports injuries or other medical conditions. Also, the great heterogeneity of the fatigue protocols, of the joint position sense measures, and of the sports practiced by the included patients, as well as the presence of many studies with a high risk of bias, represent other limitations of this study.

### Implications for practice

Although injuries in sports can be anticipated and prevented to some extent, it may not be possible to completely eliminate them. However, implementing injury prevention strategies can help to decrease both the frequency and severity of injuries [94]. Athletes who are better equipped to deal with sport-specific levels of fatigue are more likely to be able to effectively manage situations that could potentially lead to injuries [95]. It can be assumed that fatigue is a component of the injury risk profile and the appropriate program can be incorporated into training to manage fatigue [96]. Thus, injury prevention programs are recommended to include specific sports and complex cognitive movement tasks [97, 98]. One study suggests that incorporating decision-making tasks that are complex and challenging during training can help athletes develop optimal movement strategies for different types of situations, which can decrease the risk of injury. Additionally, some experts recommend training programs that simulate realistic scenarios by incorporating fatigued conditions to train athletes in managing injuries when the risk of injury is higher [98]. Furthermore, mental imagery exercises could beeffective for combating fatigue because they stimulate the central representation of movements, where movements are controlled [99].

### Methodological considerations

Due to the fact that most of the articles did not have a control group, the control group can be used in future studies. Also, most protocols are used were localized, it is better to use functional protocols in future research. In addition, it is also useful to investigated the fatigue effects on the injured participant repositioning sense.

## Conclusion

Fatigue appears to increase the active absolute angular error of the knee, ankle, and hip while not affecting the passive absolute angular error of the ankle and knee. Furthermore, fatigue has no discernible effect on the relative error of the knee. Due to insufficient studiesexamining the absolute error of ankle and hip reposition sense in the sagittal plane, further researches are required for clarification. On the whole, it appears that fatigue may increase the risk of injury by reducing proprioception. Therefore, future research could be conducted to investigate the potential impact of integrated fatigue-mitigating exercises into athletes’ training programs, with the aim of reducing the incidence of sports-related injuries.

### Supplementary Information

**Additional file 1. **Results of methodological quality.

**Additional file 2. **A Description of eligible studies.

**Additional file 3. **Funnel plot of studies worked on passive absolute error angle of the knee. Funnel plot of studies worked on active relative error angle of the knee. Funnel plot of studies worked on active absolute error angle of the ankle in the horizontal plan. Funnel plot of studies worked on passive absolute error angle of the ankle in the horizontal plan. Funnel plot of studies worked on active absolute error angle of the ankle in the sagittal plan.

**Additional file 4. **Raw data of active absolute angular error of the knee.

**Additional file 5. **Raw data of active absolute angular error of the ankle in the horizantal plane.

**Additional file 6. ** Raw data of passive absolute angular error of the ankle in horizantal plane.

**Additional file 7. **Raw data of passive absolute angular error of the knee.

**Additional file 8. **Raw data of active absolute angular error of the hip.

**Additional file 9. **Raw data of active relative angular error of the knee.

## Data Availability

The raw data and material will be available online after publishing the paper as a supplementary file 3–9 in the journal.
