# Supplementary material for: The effectiveness of fatigue on repositioning sense of lower extremities: systematic review and meta-analysis
Source: BMC Sports Sci Med Rehabil. 2024 Feb 5;16:35. doi: 10.1186/s13102-024-00820-w (PMC10840207; doi:10.1186/s13102-024-00820-w)
Supplement: Supplementary file 1 — Additional file 1. Results of methodological quality. [file 13102_2024_820_MOESM1_ESM.docx]

| **Risk of biad** | **score** | **Q22** | **Q21** | **Q20** | **Q19** | **Q18** | **Q17** | **Q16** | **Q15** | **Q14** | **Q13** | **Q12** | **Q11** | **Q10** | **Q9** | **Q8** | **Q7** | **Q6** | **Q5** | **Q4** | **Q3** | **Q2** | **Q1** | **study** |
| --- | --- | --- | --- | --- | --- | --- | --- | --- | --- | --- | --- | --- | --- | --- | --- | --- | --- | --- | --- | --- | --- | --- | --- | --- |
| high | 14/23 | 0 | 1 | 0 | 0 | 1 | 0 | 1 | 1 | 1 | 0 | 0 | 0 | 0 | 0 | 1 | 1 | 1 | 1 | 1 | 1 | 1 | 1 | Bayramoglu et al, 2007 |
| low | 23/15 | 0 | 0 | 0 | 1 | 1 | 1 | 1 | 1 | 1 | 1 | 0 | 0 | 0 | 0 | 0 | 1 | 1 | 1 | 1 | 1 | 1 | 1 | Miura et al, 2004 |
| high | 23/14 | 0 | 0 | 0 | 0 | 1 | 1 | 1 | 1 | 1 | 1 | 0 | 0 | 0 | 0 | 1 | 1 | 1 | 1 | 1 | 1 | 1 | 1 | Ju et al, 2010 |
| high | 23/13 | 0 | 0 | 0 | 0 | 1 | 1 | 1 | 1 | 1 | 1 | 0 | 0 | 0 | 0 | 0 | 1 | 1 | 1 | 1 | 1 | 1 | 1 | Ribeiro et al, 2007 |
| high | 23/14 | 0 | 0 | 0 | 0 | 1 | 1 | 1 | 1 | 1 | 1 | 0 | 0 | 0 | 0 | 0 | 1 | 1 | 2 | 1 | 1 | 1 | 1 | Ribeiro et al, 2008 |
| low | 17/23 | 0 | 1 | 0 | 0 | 1 | 1 | 1 | 1 | 1 | 1 | 0 | 1 | 0 | 0 | 1 | 1 | 1 | 2 | 1 | 1 | 1 | 1 | Franco and Reyes, 2017 |
| high | 23/14 | 0 | 1 | 0 | 0 | 1 | 1 | 1 | 1 | 1 | 1 | 0 | 0 | 0 | 0 | 0 | 1 | 1 | 1 | 1 | 1 | 1 | 1 | Han et al, 2014 |
| low | 23/15 | 0 | 1 | 0 | 0 | 1 | 1 | 1 | 1 | 1 | 1 | 0 | 0 | 0 | 0 | 1 | 1 | 1 | 1 | 1 | 1 | 1 | 1 | Ribeiro et al, 2011 |
| high | 23/13 | 0 | 1 | 0 | 0 | 0 | 1 | 1 | 1 | 1 | 1 | 0 | 0 | 0 | 0 | 0 | 1 | 1 | 1 | 1 | 1 | 1 | 1 | Givoni et al, 2007 |
| high | 23/13 | 0 | 0 | 0 | 0 | 1 | 1 | 1 | 1 | 1 | 1 | 0 | 0 | 0 | 0 | 1 | 1 | 1 | 1 | 0 | 1 | 1 | 1 | Seo et al, 2020 |
| low | 23/15 | 0 | 0 | 0 | 0 | 1 | 1 | 1 | 1 | 1 | 1 | 0 | 0 | 0 | 0 | 1 | 1 | 1 | 2 | 1 | 1 | 1 | 1 | Hisham et al, 2019 |
| high | 23/13 | 0 | 0 | 0 | 1 | 1 | 1 | 1 | 1 | 1 | 1 | 0 | 0 | 0 | 0 | 1 | 0 | 1 | 0 | 1 | 1 | 1 | 1 | Ghanbari et al,  2014 |
| high | 23/14 | 0 | 0 | 0 | 1 | 1 | 1 | 1 | 1 | 1 | 1 | 0 | 0 | 0 | 0 | 1 | 1 | 1 | 0 | 1 | 1 | 1 | 1 | choi and Chung-hwi, 2003 |
| high | 14/23 | 0 | 0 | 0 | 0 | 1 | 1 | 1 | 1 | 1 | 1 | 0 | 0 | 0 | 0 | 1 | 1 | 1 | 1 | 1 | 1 | 1 | 1 | Changela et al, 2012 |
| low | 16/23 | 1 | 0 | 0 | 0 | 1 | 1 | 1 | 1 | 1 | 1 | 0 | 0 | 0 | 0 | 1 | 1 | 2 | 2 | 1 | 0 | 1 | 1 | Niederseer et al,2014 |
| high | 14/23 | 0 | 0 | 0 | 0 | 1 | 0 | 1 | 1 | 1 | 1 | 0 | 0 | 0 | 0 | 1 | 1 | 1 | 2 | 1 | 1 | 1 | 1 | Dieling et al,2014 |
| low | 17/23 | 1 | 1 | 0 | 0 | 1 | 1 | 1 | 1 | 1 | 1 | 0 | 0 | 0 | 0 | 1 | 1 | 1 | 2 | 1 | 1 | 1 | 1 | Salgado et al, 2015 |
| low | 16/23 | 0 | 1 | 0 | 0 | 1 | 1 | 1 | 1 | 1 | 1 | 0 | 0 | 0 | 0 | 1 | 1 | 1 | 2 | 1 | 1 | 1 | 1 | Gear et al, 2011 |
| low | 16/23 | 1 | 1 | 0 | 1 | 1 | 1 | 1 | 1 | 1 | 1 | 0 | 0 | 0 | 0 | 1 | 1 | 1 | 0 | 1 | 1 | 1 | 1 | Négyesi et al,2021 |
| low | 17/23 | 1 | 1 | 0 | 0 | 1 | 1 | 1 | 1 | 1 | 1 | 0 | 0 | 0 | 0 | 1 | 1 | 1 | 2 | 1 | 1 | 1 | 1 | Steib et al,2013 |
| low | 16/23 | 0 | 1 | 0 | 0 | 1 | 1 | 1 | 1 | 1 | 1 | 0 | 0 | 0 | 0 | 1 | 1 | 1 | 2 | 1 | 1 | 1 | 1 | Huston et al,2005 |
| low | 16/23 | 0 | 1 | 0 | 0 | 1 | 1 | 1 | 1 | 1 | 1 | 0 | 0 | 0 | 0 | 1 | 1 | 1 | 2 | 1 | 1 | 1 | 1 | Sandrey and kent,2008 |
| high | 14/23 | 0 | 0 | 0 | 0 | 1 | 1 | 1 | 1 | 1 | 1 | 0 | 0 | 0 | 0 | 1 | 1 | 1 | 1 | 1 | 1 | 1 | 1 | Jahjah et al,2018 |
| low | 17/23 | 0 | 1 | 0 | 1 | 1 | 1 | 1 | 1 | 1 | 1 | 0 | 0 | 0 | 0 | 1 | 1 | 1 | 2 | 1 | 1 | 1 | 1 | Eftekhari et al,2018 |
| low | 16/23 | 0 | 0 | 0 | 0 | 1 | 1 | 1 | 1 | 1 | 1 | 1 | 0 | 0 | 0 | 1 | 1 | 1 | 2 | 1 | 1 | 1 | 1 | Naderian et al,2018 |
| high | 14/23 | 0 | 0 | 0 | 0 | 1 | 1 | 1 | 1 | 1 | 1 | 0 | 0 | 0 | 0 | 1 | 1 | 1 | 1 | 1 | 1 | 1 | 1 | Mohammadi Bazneshin et al, 2015 |
| low | 15/23 | 0 | 0 | 0 | 0 | 1 | 1 | 1 | 1 | 1 | 1 | 0 | 0 | 0 | 0 | 1 | 1 | 1 | 2 | 1 | 1 | 1 | 1 | Hussieni et al,2021 |
| low | 16/23 | 0 | 0 | 0 | 1 | 1 | 1 | 1 | 1 | 1 | 1 | 0 | 0 | 0 | 0 | 1 | 1 | 1 | 2 | 1 | 1 | 1 | 1 | Marks and Quinney ,1993 |
| low | 15/23 | 0 | 0 | 0 | 1 | 0 | 1 | 1 | 1 | 1 | 1 | 0 | 0 | 0 | 0 | 1 | 1 | 1 | 2 | 1 | 1 | 1 | 1 | Mark, 1994 |
| low | 15/23 | 0 | 1 | 0 | 0 | 1 | 1 | 1 | 1 | 1 | 1 | 0 | 0 | 0 | 0 | 1 | 1 | 1 | 1 | 1 | 1 | 1 | 1 | Skinner et al, 1986 |
| high | 13/23 | 0 | 1 | 0 | 0 | 0 | 1 | 1 | 1 | 1 | 1 | 0 | 0 | 0 | 0 | 0 | 1 | 1 | 1 | 1 | 1 | 1 | 1 | Allen et al, 2010 |
| high | 14/23 | 0 | 0 | 0 | 0 | 1 | 1 | 1 | 1 | 1 | 1 | 0 | 0 | 0 | 0 | 0 | 1 | 1 | 2 | 1 | 1 | 1 | 1 | Ghahremani et al, 2017 |
| high | 14/23 | 0 | 0 | 0 | 0 | 1 | 1 | 1 | 1 | 1 | 1 | 0 | 0 | 0 | 0 | 1 | 1 | 1 | 1 | 1 | 1 | 1 | 1 | Arvin et al 2015, |
| high | 12/23 | 0 | 0 | 0 | 0 | 1 | 1 | 1 | 1 | 1 | 1 | 0 | 0 | 0 | 0 | 0 | 1 | 1 | 1 | 1 | 0 | 1 | 1 | Forestier et al,2006 |
| high | 12/23 | 0 | 0 | 0 | 0 | 0 | 0 | 1 | 1 | 1 | 1 | 0 | 0 | 0 | 0 | 1 | 1 | 1 | 1 | 1 | 1 | 1 | 1 | Lin et al,2008 |
| low | 16/23 | 0 | 0 | 0 | 1 | 1 | 1 | 1 | 1 | 1 | 1 | 0 | 1 | 0 | 0 | 0 | 1 | 1 | 2 | 1 | 1 | 1 | 1 | Mohammadi et al, 2010 |
| high | 11/23 | 0 | 0 | 0 | 0 | 1 | 1 | 0 | 1 | 0 | 1 | 0 | 0 | 0 | 0 | 0 | 1 | 1 | 1 | 1 | 1 | 1 | 1 | Ahn et al,2015 |
| low | 15/23 | 0 | 0 | 0 | 0 | 1 | 1 | 1 | 1 | 1 | 1 | 0 | 0 | 0 | 0 | 1 | 1 | 1 | 2 | 1 | 1 | 1 | 1 | Tomar et al, 2014 |
| high | 12/23 | 0 | 0 | 0 | 0 | 1 | 1 | 1 | 1 | 1 | 1 | 0 | 0 | 0 | 0 | 1 | 1 | 1 | 1 | 1 | 0 | 1 | 0 | Draz et al 2013 |
| low | 17/23 | 1 | 1 | 0 | 0 | 1 | 1 | 1 | 1 | 1 | 1 | 0 | 0 | 0 | 0 | 1 | 1 | 1 | 2 | 1 | 1 | 1 | 1 | Bersotti et al, 2022 |
| low | 17/23 | 0 | 1 | 0 | 0 | 0 | 1 | 1 | 1 | 1 | 1 | 1 | 1 | 0 | 0 | 1 | 1 | 1 | 2 | 1 | 1 | 1 | 1 | Hong et al, 2022 |
| high | 14/23 | 0 | 0 | 0 | 0 | 1 | 1 | 1 | 1 | 1 | 1 | 0 | 0 | 0 | 0 | 1 | 1 | 1 | 1 | 1 | 1 | 1 | 1 | Ju et al 2011 |
| low | 15/23 | 0 | 0 | 0 | 0 | 1 | 1 | 1 | 1 | 1 | 1 | 0 | 0 | 0 | 0 | 1 | 1 | 1 | 2 | 1 | 1 | 1 | 1 | Sharma et al,2023 |

Supplementary material 1: Results of methodological quality
