# Supplementary material for: The effectiveness of fatigue on repositioning sense of lower extremities: systematic review and meta-analysis
Source: BMC Sports Sci Med Rehabil. 2024 Feb 5;16:35. doi: 10.1186/s13102-024-00820-w (PMC10840207; doi:10.1186/s13102-024-00820-w)
Supplement: Supplementary file 2 — Additional file 2. A Description of eligible studies. [file 13102_2024_820_MOESM2_ESM.docx]

| **Study** | **Study design** | **Subjects** | |  |  |  |  |  |  |
| --- | --- | --- | --- | --- | --- | --- | --- | --- | --- |
|  |  | **Sample description** | **Sample Size**  **(Men/Women)**  **(Age ± SD)** | **Exercise Intervention Program** | **Control Intervention** | **Measured Variables** | **Main Outcomes** | **Downs and Black score** | **Risk of biad** |
| Bayramoglu et al, 2007 | Cross-sectional | healthy volunteers | E=30 (5/25)  (57.86±9.22) | 5min Cycling on a cycle ergometer |  | passive knee JPS (AAE, 45), isokinetic dynamometer | AAE did not different between pre and post exercise. | 14/23 | high |
| Miura et al, 2004 | Experimental controlled | Healthy volunteers | E=27 (all male) (22.2) | 60 consecutive maximum concentric contractions of the knee extensors and flexors on the isokinetic dynamometer |  | Active knee JPS(AAE, betwwen10-80), photogrammetry | After local load no significant change in AAE was seen. | 23/15 | low |
| Ju et al, 2010 | Quasi-experimental | healthy volunteers | E=15(8/7)(24.4±1.5 ) | 60 Maximum concentric and eccentric contraction of the quadriceps |  | passive and active Knee JPS (AAE,RAE, between 10-80), self-design device  . | statistically significant increase in AAE following repetitive active movement was seen, but not in RAE | 23/14 | high |
| Ribeiro et al, 2007 | Quasi-experimental | healthy subjects | E=60 (all male) (69.81 ± 3.92) | Warm up + 30 consecutive maximal gravity corrected concentric contractions of the knee extensors and flexors |  | Active knee JPS ( AAE and RAE, between 40-60), photogrammetry | RAE and AAE were increased significantly. | 23/13 | high |
| Ribeiro et al, 2008 | Cross-sectional study | Elite volleyball players from the Portuguese national team | E=17 (all Female) (18.99±4.2) | 25-min warm up + volleyball match of five sets to mimic competition activity |  | Active knee JPS (AAE,RAE, between 40-60), photogrammetry | A significant increase in absolute and relative angular errors was detected. | 23/14 | high |
| Franco and Reyes, 2017 | Randomized controlled trial | sprinters from an athletic club | E: 16 (all male) (25.0 ± 3.6)  C= 16 (all male) (24.7 ±3.4) | plyometric exercise | No exercise | Active knee JPS (AAE,RAE, 50 (40-60), photogrammetry | RAE, worsened compared to the control group and to the values AAE did not show significant differences. | 17/23 | low |
| Han et al, 2014 | Cross-sectional study | healthy adults | E= 15 (all male) (21.87 ± 0.83) | Three sets of 10 squats with 30 seconds of rest between sets. |  | Active knee JPS (AAE, 30°, 45°,60°), digital goniometer | AAE increased after quadriceps fatigue. | 23/14 | high |
| Ribeiro et al, 2011 | within-subjects repeated-measures | healthy subjects | E= 40 (all male) (22.1 ± 3.0) | 30 consecutive maximal gravity corrected concentric / eccentric contractions of the knee extensors |  | Active knee JPS (AAE,RAE, 60°), isokinetic dynamometer | Increased the AAE but no changes were observed in the RAE. | 23/15 | low |
| Givoni et al, 2007 | Quasi-experimental | healthy subjects | Eccentric exercise**:** 8(2/6)  ( 20.2±1.0 )  Concentric exercise**:** 10(2/8)  ( 20.0±1.8) | **Eccentric exercise**: walking down six flights of stairs, two steps at a time, until they had completed 11 circuits representing a total of 792 steps.  **Concentric exercise**:  walking up six flights of stairs, two steps at a time, until they had completed this 11 times, representing 792 steps |  | Active knee JPS (RAE, 50), potentiometer | RAE increased significantly after Eccentric and Concentric exercise. | 23/13 | high |
| Seo et al, 2020 | Pre-post test | Taekwondo athletes | Kyorugi=14  Poomsae= 14 | E1= Kyorugi (full-contact sparring) training  E2= Poomsae training |  | Active knee JPS ( RAE, 40), Goniometer and photogrammetry | Studies have shown no significant impact on the RAE caused by muscle fatigue induced by Poomsae and Kyorugi | 23/13 | high |
| Hisham et al, 2019 | One group pretest/posttest cross section | volleyball players | E=25(all Female) (20±2) | several submaximal repetitions of in-version-eversion + performing continuous inversion-eversion movements, until the force production of the personal muscles decrease below the half of maximal peak torque |  | Passive ankle JPS ( AAE, 20 degree of inversion), isokinetic dynamometer | There was no statistical significant difference in AAE before and after peroneal muscles fatigue | 23/15 | low |
| Ghanbari et al,  2014 | Cross-sectional | healthy subjects | E=40 (all male) (23.9±2.3 ) | Open-chain group: isometric contraction of the tibialis anterior equal to 70% of MVC and hold the contraction as long as the dynamometer showed a number above 50% of MVC .  .  closed-chain group:  It was similar to that of the open-chain group except that the subjects were standing on a platform, off the ground. They were asked to dorsiflexion their ankle with the dynamometer attached to a hook on the ground. |  | Passive and active ankle JPS,  (AA, 21° plantar flexion), pedal goniometer | There was significant decrease in subjects’ abilities to recognize active  and passive repositioning of their ankle after a fatigue protocol | 23/13 | high |
| choi and Chung-hwi, 2003 | Quasi-experimental | healthy women | E= 40 (all female) (20.9)  10 participant in each group | performing isokinetic pedaling for 50 seconds at different exercise intensities  Experimental group 1: 10% intensity Experimental group 2: 30% intensity  Experimental group 3: 50% intensity Experimental group 4: 70% intensity |  | Active knee JPS (AAE, 135), isokinetic dynamometer | Significant differences were found between group 4 and group 1, 2 and 3, but significant differences were not found between groups 1, 2 and 3. | 23/14 | high |
| Changela et al, 2012 | Observational study | healthy subjects | E: 30 (21/9) (18-30) | cycling on a static cycle as fast as possible, Fatigue was induced in the subjects by cycling When subjects reached up to level of exceeding 60% of predicted HRmax and a level of exertion of 14-17 on the RPE scale |  | Active knee JPS (AAE, 30), goniometer and photogrammetry | After inducing fatigue, AAE was increased. | 14/23 | high |
| Niederseer et al,2014 | Single group pre-post | professional handball players | E=19 (all male) ( 22.3 ± 4.9) | a simulated handball match |  | Active hip JPS (AAE, 30 internal and external rotation)  Active knee JPS (extensions), inclinometer | Hips JPS decreased significantly but knees were not affected by the simulated handball match. | 16/23 | low |
| Dieling et al,2014 19 | Quasi-experimental | E= ballet dancer  C= non-dancer | E=13(3/10)  (27.5 ± 6.8)  C=13 (4/9)  (25.7 ± 3.7) | maximal isokinetic knee flexion and extension contractions | maximal isokinetic knee flexion and extension contractions | Passive knee JPS (AAE, 30°, 45°, and 60), isokinetic dynamometer | AAE was not affected by muscle fatigue in dancers. | 14/23 | high |
| Salgado et al, 2015 | Cross-sectional | semi-professional football players | E=40 (all male)( 25.9 ± 4.6) | competitive football match |  | active knee JPS (AAE, RAE, between 40-60), photogrammetry | AAE and RAE increased significantly after fatigue. | 17/23 | low |
| Gear et al, 2011 | Repeated  measures | Participants recruited from NCAA  Division III basketball and soccer programs | E= 18(8/10)  Female=( 19.50 ± 1.18)  male= (20.88 ± 1.25) | Knee extension and flexion isokinetic exercise until torque output was, 70of the peak hamstring torque for three consecutive repetitions. |  | Active knee JPS (AAE, 45°), isokinetic dynamometer | There weren't significant difference between the pre-test and following 70% of peak hamstring torque. | 16/23 | low |
| Négyesi et al,2021 | Quasi-experimental | healthy adults | E=8 (25.5 ± 4) | 100 continuous maximal voluntary  eccentric activations with the knee extensors |  | Active knee JPS (AAE,RAE, 58),isokinetic dynamometer | AAE increased post-intervention. | 16/23 | low |
| Steib et al,2013 | Quasi-experimental | athletes  (Handball, volleyball, basketball, soccer). | E=19 (13/6)  (23.32±3.79 | Run as long as possible until complete exhaustion, and verbal encouragement was provided towards the end of the run. |  | Passive ankle JPS (AAE, inversion 10°), isokinetic dynamometer | Fatiguing exercise did not effect on proprioception. | 17/23 | low |
| Huston et al,2005 | 2 × 2 factorial design | healthy subjects | E=20 (10/10)  (21.75 ± 1.48) | stand as long as possible with both feet in the plantar-flexed position, sustaining an isometric plantar flexion of the 2 legs.( gastrocnemius and soleus fatigue) |  | Passive ankle JPS (AAE, 20° of plantar flexion), joint position sense device | There was no significant difference after fatigue. | 16/23 | low |
| Sandrey and kent,2008 | 2 × 2 factorial design | healthy subjects | E=40 (16/24)  (20.10 ± 2.02) | Muscular fatigue was assumed when subjects were unable to complete 3 consecutive repetitions for both concentric and eccentric eversion at 50% or greater of their maximum joint torque. |  | passive ankle JPS (AAE, 20° of inversion), joint position sense device | AAE was significantly higher after fatigue. | 16/23 | low |
| Jahjah et al,2018 | Randomized controlled trial | healthy subjects | E=17(26,8 ± 2,6) | 30 consecutive maximal concentric /concentric contractions of the ankle evertors and invertors |  | Active and passive ankle JPS (AAE, 15°inversion),  isokinetic dynamometer | These results showed an increase in AAE after muscle fatigue. | 14/23 | high |
| Eftekhari et al,2018 | Semi-experimental | healthy athletic | E=10 (all female) (21/75±0/46) | Jogging +15 minutes dynamic stretching + bipedal and single-legged jumps in Different directions Using a 20 cm step |  | Active and passive knee JPS ( AAE ,60), isokinetic dynamometer | JPS was not significantly after fatigue. | 17/23 | low |
| Naderian et al,2018 | Semi-experimental | futsal students of Isfahan University | E=20 (all female)( 20 ± 1. 8) | Bangsbo modified futsal fatigue protocol |  | Active ankle JPS (AAE, 15degree inversion and eversion), isokinetic dynamometer | AAE was significantly increased after fatigue. | 16/23 | low |
| Mohammadi Bazneshin et al, 2015 | Pretest-posttest | healthy young subjects | E=34 (all male) (22.15±1.97) | Quadriceps muscle fatigue firstly with 70% of the maximum quadriceps isometric force then 50% of maximum isometric force of the quadriceps |  | Active knee JPS (AAE, 45), digital inclinometer | Quadriceps muscle fatigue causes a significant increase in the AAE. | 14/23 | high |
| Marks and Quinney ,1993 | Randomized controlled | healthy sedentary women | E= 8 (all female) (19.7±1.2  C=8 (all female) (22.3±3.1) | 20 consecutive concentric and eccentric contractions of the quadriceps femoris muscle | No exercise | Active knee JPS (AAE,45 to 75), isokinetic  dynamometer | the mean of AAE by the experimental group remained unchanged after the exercises | 15/23 | low |
| Mark, 1994 | Randomized controlled | healthy sedentary  women | E=6 (all female) (18-30)  C=6 (all female) (18-30) | 20 reciprocal maximal concentric and eccentric isokinetic quadriceps contractions | No exercise | Active knee JPS (AAE, RAE, 45 - 75 ), isokinetic dynamometer | There is not significant for the analysis of the differential in mean AAE. | 16/23 | low |
| Skinner et al, 1986 | Quasi-experimental | healthy subject | E=11(all male) | 35 knee flexion/extension repetitions |  | Active knee JPS ( AAE, 5 to 25) , | Absolute error increased significantly after fatigue. | 15/23 | low |
| Allen et al, 2010 | Quasi-experimental | young adults | E= 10 | Concentric contractions of knee flexors in the manner of a knee  Curl. |  | Active knee JPS (RAE, 45), potentiometer | RAE after exercise was significantly different from RAE before exercise. | 15/23 | low |
| Ghahremani et al, 2017 | Pre-post test | karate athletes participating in Iranian national league | E= 10 (all male) (21.73±1.83) | Sorensen test |  | Active knee JPS (AAE, 60),  Active hip JPS (AAE, 30 abduction, 30 flexion )  goniometer and photogrammetry | Erector spine muscle fatigue did not have a significant effect on knee JPS. | 13/23 | high |
| Arvin et al 2015, | Quasi-experimental | healthy volunteers | E=17(5/12) (73.2±7.7) | Hip adduction with weight of 20% of the participant’s total leg moment as estimated from anthropometrical data. |  | Active hip JPS (AAE, 10-40abduction), Optotrak | Hip position sense was also affected by fatigue, as indicated by an increased relative and absolute error | 14/23 | high |
| Forestier et al,2006 | Quasi-experimental | healthy adults | E=10 (all male) (20-27) | Maintain a workload equal to 70% of their MVC in series of 40 s with 40 s rest after each trial. The fatigue level was reached when subjects were not able to maintain the workload for more than 15 s. |  | Active ankle JPS (AAE, 15 dorsi and plantar flexion ), potentiometers | There was no significant difference in AAE before and after fatigue. | 14/23 | high |
| Lin et al,2008 | Cross-sectional before-after study with repeated measures | Subject without proprioception defective | E=30(22.8 ± 1.0) | Muscle fatigue of ankle invertors and evertors was defined as occuring when the maximal isokinetic torque declined to below 50% of the peak torque |  | passive ankle JPS ( AAE, 10 inversion), Isokinetic Dynamometer | Muscle fatigue increased the AAE. | 12/23 | high |
| Mohammadi et al, 2010 | Controlled laboratory | soccer players | E= 32 (all male)  ( 24.7 6 1.3)  E1=16  E2=16 | E1= 45 minutes of playing soccer  E2= inversion/eversion workload equal to 70% of their MVC for as long as possible. Termination of the exercise occurred when subjects could no longer maintain 50%  of MVC for approximately 3 seconds |  | Active and passive ankle JPS( AAE, 15 inversion), isokinetic dynamometer | The acuity of the ankle JPS is reduced subsequent to a fatigue protocol in both groups. | 12/23 | high |
| Ahn et al,2015 | Randomized controlled  cross-sectional | healthy volunteers | E=15(all female) (30.26 ± 3.41) | Subject lie prone with knee was flexed 90 then pushed digital MMT located over the ankle joint lasting for 5 s each and 2 s breaks until the peak torque dropped to 50% of the initial value |  | Active knee JPS (AAE,RAE, 135), digital inclinometer | There was no significant difference in AAE and RAE before and after fatigue. | 16/23 | low |
| Draz et al 2013 | Pre-post test | normal subjects | E=100 (19.18 ±.7) | isokinetic lumbar flexion and extension with maximal effort until the lumbar extensor peak torque dropped below 50% for 3 consecutive repetitions |  | Active knee JPS (AAE, 45), isokinetic dynamometer | AAE was significantly increased after fatigue protocol | 11/23 | high |
| Tomar et al, 2014 | Quasi-experimental | healthy college students | E= 100(50/50) | stand on their tiptoes until they could no longer hold such a position |  | Active ankle JPS (AAE, 20 plantar), Electric goniometer | There was no significant change in absolute error in an angle reproduction test at 20° of plantar flexion. | 15/23 | low |
| Hussieni et al,2021 | Cross-sectional | volleyball players | E= 25 (all female) (20±2) | repeated eversion contractions above 70% of the predetermined  maximum voluntary contraction until 3 successive contractions  dropped below 50% of the predetermined maximum level. |  | Passive ankle JPS (AAE, 10 inversion) ,  isokinetic dynamometer | No Significant differences were found between fatigue and non-fatigue. | 12/23 | high |
| Ju et al 2011 | Quasi-experimental | Healty subjects | E=16(8/8)(22.1±2.63) | 30 times repeated passive knee  movement in knee angular velocities 90°/s, |  | Active Knee JPS (AAE, between 30-60), self-design device  . | The results revealed a decrease in error scores in active repositioning | 17/23 | low |
| Bersotti et al, 2022 | Cross-sectional | physically active person | E= 15 (all male) (23.4±3.8) | concentric maximum voluntary contractions of knee joint until the generated peak torque was equal to or less than 50% |  | Active knee JPS (AAE, 60), isokinetic dynamometer | Absolute error was not significantly  affected by muscle fatigue. | 17/23 | low |
| Hong et al, 2022 | Quasi-experimental | physically active person | E=12 (6/6) ( 22.8±2.2) | Countermovement jump on a force plate until Borg rating of perceived exertion (RPE) reached level 17 or higher |  | Active knee JPS (AAE, 45), goniometer | No significant differences were found after fatigue protocol | 14/23 | high |
| Sharma et al,2023 | experimental | recreational athletes | E=25 (18-25) | 20-metre shuttle test |  | Active knee JPS (AAE, 30), goniometer | the knee joint proprioception was significantly reduced after fatigue | 15/23 | low |

supplementary 2: A Description of Eligible Studies

E: Experimental group, C: Control group, JPS: Joint Position Sense, AAE: absolute angular errorو RAE: relative angular error, CKC: close kinematic chain
