# Supplementary material for: The effectiveness of fatigue on repositioning sense of lower extremities: systematic review and meta-analysis
Source: BMC Sports Sci Med Rehabil. 2024 Feb 5;16:35. doi: 10.1186/s13102-024-00820-w (PMC10840207; doi:10.1186/s13102-024-00820-w)
Supplement: Supplementary file 3 — Additional file 3. Funnel plot of studies worked on passive absolute error angle of the knee. Funnel plot of studies worked on active relative error angle of the knee. Funnel plot of studies worked on active absolute error angle of the ankle in the horizontal plan. Funnel plot of studies worked on passive absolute error angle of the ankle in the horizontal plan. Funnel plot of studies worked on active absolute error angle of the ankle in the sagittal plan. [file 13102_2024_820_MOESM3_ESM.docx]

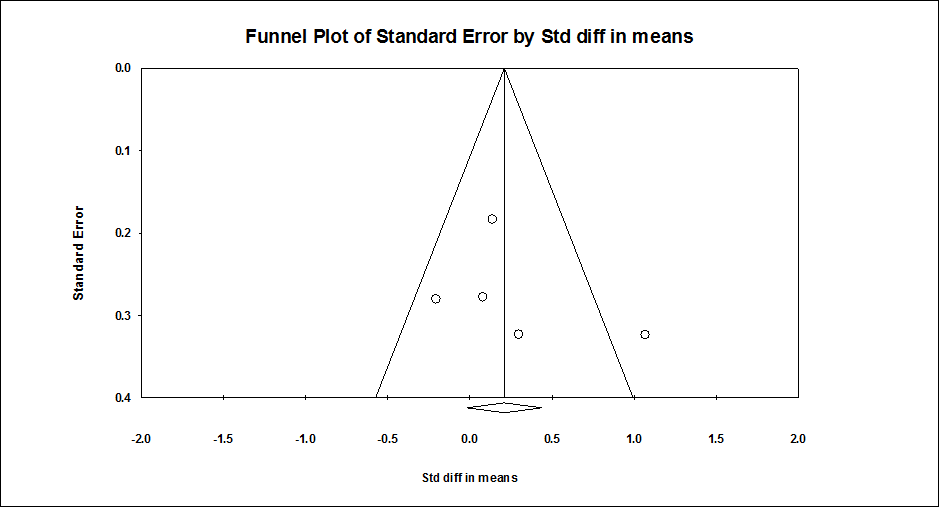


Funnel plot of studies worked on passive absolute error angle of the knee


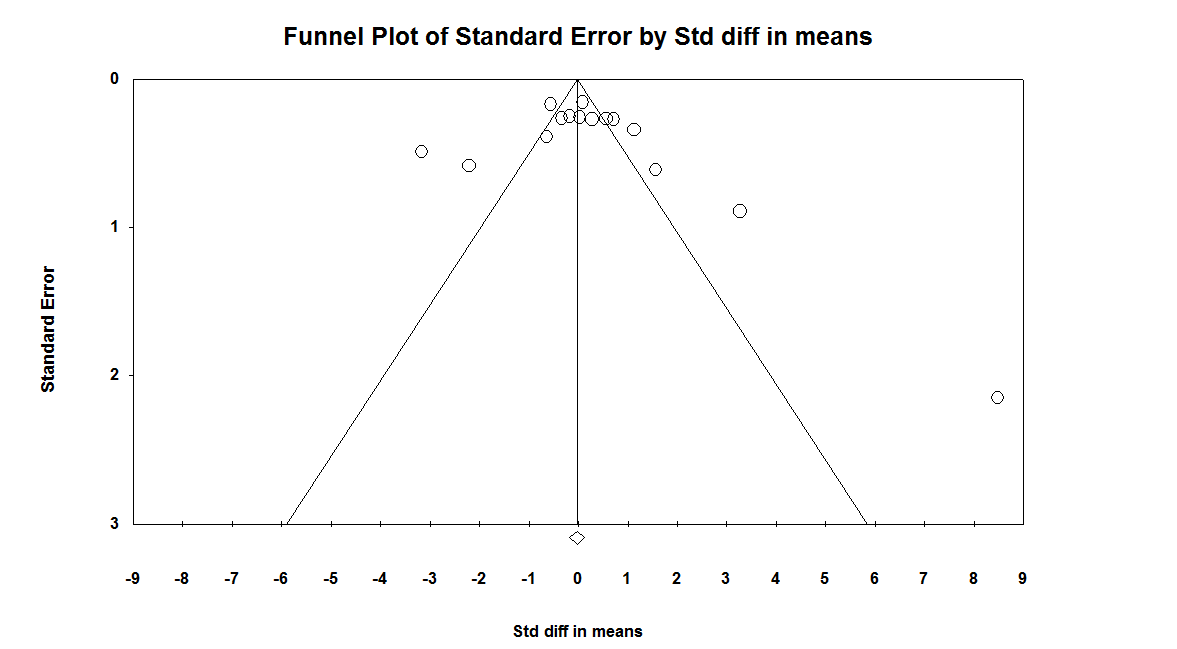


Funnel plot of studies worked on active relative error angle of the knee


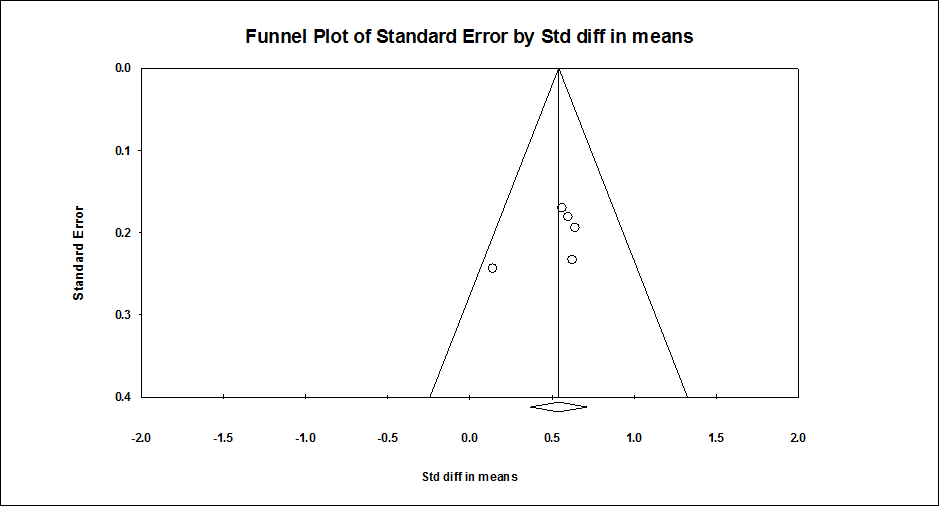


Funnel plot of studies worked on active absolute error angle of the ankle in the horizontal plan


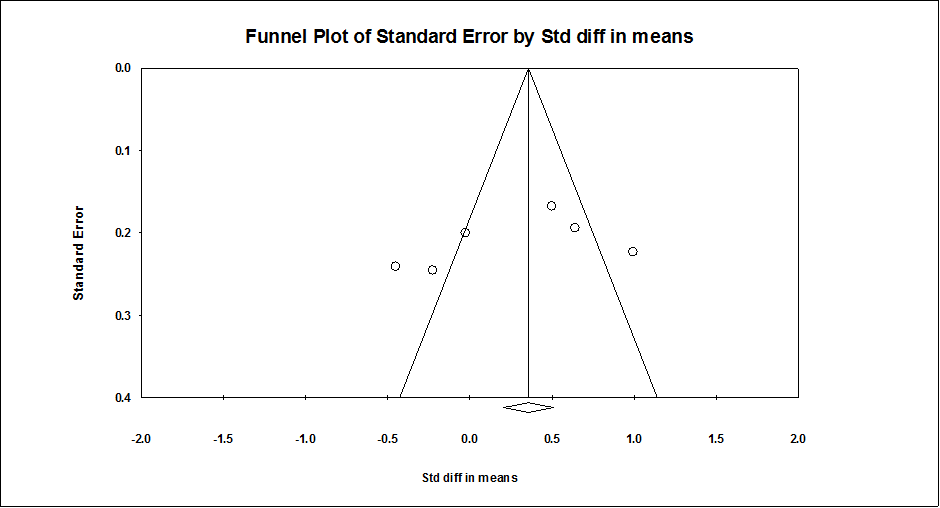


Funnel plot of studies worked on passive absolute error angle of the ankle in the horizontal plan


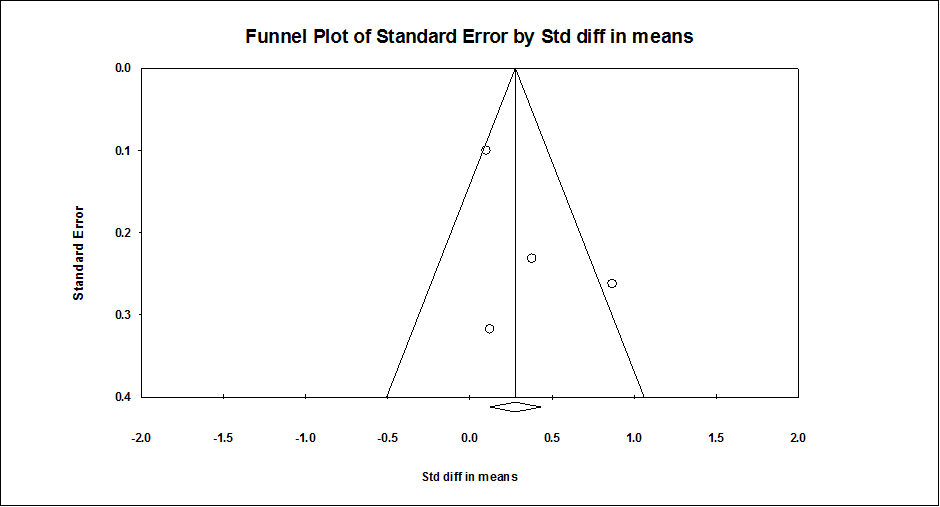


Funnel plot of studies worked on active absolute error angle of the ankle in the sagittal plan
